# Achieving enhanced diagnostic precision in endometrial lesion analysis through a data enhancement framework

**DOI:** 10.3389/fonc.2024.1440881

**Published:** 2024-10-15

**Authors:** Yi Luo, Meiyi Yang, Xiaoying Liu, Liufeng Qin, Zhengjun Yu, Yunxia Gao, Xia Xu, Guofen Zha, Xuehua Zhu, Gang Chen, Xue Wang, Lulu Cao, Yuwang Zhou, Yun Fang

**Affiliations:** ^1^ Medical Engineering Cross Innovation Consortium, Yangtze Delta Region Institute (Quzhou), University of Electronic Science and Technology of China, Quzhou, Zhejiang, China; ^2^ Medical Engineering Cross Innovation Consortium, The Quzhou Affiliated Hospital of Wenzhou Medical University, Quzhou People’s Hospital, Quzhou, Zhejiang, China; ^3^ School of Computer Science and Engineering, University of Electronic Science and Technology of China, Chengdu, Sichuan, China; ^4^ Department of Ultrasound, The Quzhou Affiliated Hospital of Wenzhou Medical University, Quzhou People’s Hospital, Quzhou, Zhejiang, China; ^5^ Department of Ultrasound, Kaihua County People’s Hospital, Quzhou, Zhejiang, China; ^6^ Department of Ultrasound, The Second People’s Hospital of Quzhou, Quzhou, Zhejiang, China; ^7^ Department of Ultrasound, Changshan County People’s Hospital, Quzhou, Zhejiang, China; ^8^ Department of Ultrasound, People’s Hospital of Quzhou Kecheng, Quzhou, Zhejiang, China; ^9^ Department of Ultrasound, Quzhou Maternal and Child Health Care Hospital, Quzhou, Zhejiang, China; ^10^ Department of Pathology, The Quzhou Affiliated Hospital of Wenzhou Medical University, Quzhou People’s Hospital, Quzhou, Zhejiang, China

**Keywords:** deep learning, data enhancement framework, endometrial cancer, ultrasonography, diagnosis

## Abstract

**Objective:**

The aim of this study was to enhance the precision of categorization of endometrial lesions in ultrasound images via a data enhancement framework based on deep learning (DL), through addressing diagnostic accuracy challenges, contributing to future research.

**Materials and methods:**

Ultrasound image datasets from 734 patients across six hospitals were collected. A data enhancement framework, including image features cleaning and soften label, was devised and validated across multiple DL models, including ResNet50, DenseNet169, DenseNet201, and ViT-B. A hybrid model, integrating convolutional neural network and transformer architectures for optimal performance, to predict lesion types was developed.

**Results:**

Implementation of our novel strategies resulted in a substantial enhancement in model accuracy. The ensemble model achieved accuracy and macro-area under the receiver operating characteristic curve values of 0.809 of 0.911, respectively, underscoring the potential for use of DL in endometrial lesion ultrasound image classification.

**Conclusion:**

We successfully developed a data enhancement framework to accurately classify endometrial lesions in ultrasound images. Integration of anomaly detection, data cleaning, and soften label strategies enhanced the comprehension of lesion image features by the model, thereby boosting its classification capacity. Our research offers valuable insights for future studies and lays the foundation for creation of more precise diagnostic tools.

## 1 Introduction

Patients with endometrial cancer, otherwise referred to as cancer of the uterine body, have a highly variable prognosis; crucially, the survival rate can be significantly improved through early detection and diagnosis (1, 2). In clinical practice, patients with postmenopausal bleeding are generally diagnosed through various means, including imaging, pathological examination, and serum tumor markers (3, 4). Magnetic resonance imaging (MRI) and computed tomography (CT) are relatively accurate imaging methods, but are expensive and CT poses significant radiation hazards. Further, although curettage and hysteroscopy are key steps in the diagnostic process, they are somewhat invasive for patients. In contrast, ultrasound examination is convenient, non-invasive, inexpensive, and repeatable, and is often used as a first-line diagnostic tool for endometrial lesions (5, 6). Ultrasonography is also an important means of large-scale asymptomatic population screening, where early detection of endometrial cancer by large-scale screening can significantly improve patient prognosis (7). Nevertheless, since physical condition and disease state vary in each patient, there is currently no universal diagnostic indicator for endometrial cancer (4). Additionally, the accuracy of ultrasound examination is affected by factors including the technical ability of medical personnel and environmental noise. Reznak et al. found that the success rate of ultrasound examination in predicting polyps is 65.1%, and that it has limited predictive value when used alone (8). Therefore, there is an urgent need for an auxiliary screening method that can effectively improve the accuracy of ultrasound examination in diagnosing endometrial cancer.

In recent years, artificial intelligence, particularly deep learning (DL), has made significant progress in medical image recognition (9–11). For instance, numerous developmental directions have emerged in the application of deep learning for the diagnosis of endometrial lesions. Based on MRI images, DL models can automatically locate, segment, and measure the degree of muscle infiltration of endometrial cancer (12–15); however, DL research based on ultrasound images is relatively scarce. Hu et al. (16) and Liu et al. (17) each proposed endometrial thickness measurement models based on transvaginal ultrasound (TVUS) images; however, these models cannot be directly applied to endometrial lesion classification. Other features in ultrasound images, such as uniformity of endometrial echo and blood flow signals, are also crucial for distinguishing benign and malignant endometrial lesions (18, 19). Further, DL also performs poorly in the task of ultrasound image classification. Raimondo et al. (20) used a DL model to diagnose adenomyosis based on TVUS images, and the results indicated that the diagnostic accuracy of the DL model was lower than that of general ultrasound doctors, although it had higher specificity in identifying healthy uteruses and reducing overdiagnosis. Therefore, we sought to improve model learning and utilization of various ultrasound image features using DL methods to enhance endometrial lesion classification accuracy.

In this study, we developed a DL model for automatic identification of endometrial lesions using an innovative combination comprising multi-stage anomaly detection, a data cleaning process, and a soft label strategy, to improve model understanding of lesion image features and enhance its classification ability. Our experiments explored the relationships among lesion features, models, and different degrees of softening ( τ ). Final accuracy was also enhanced through integration of several different models.

## 2 Materials and methods

### 2.1 Patients

**Figure 1:**
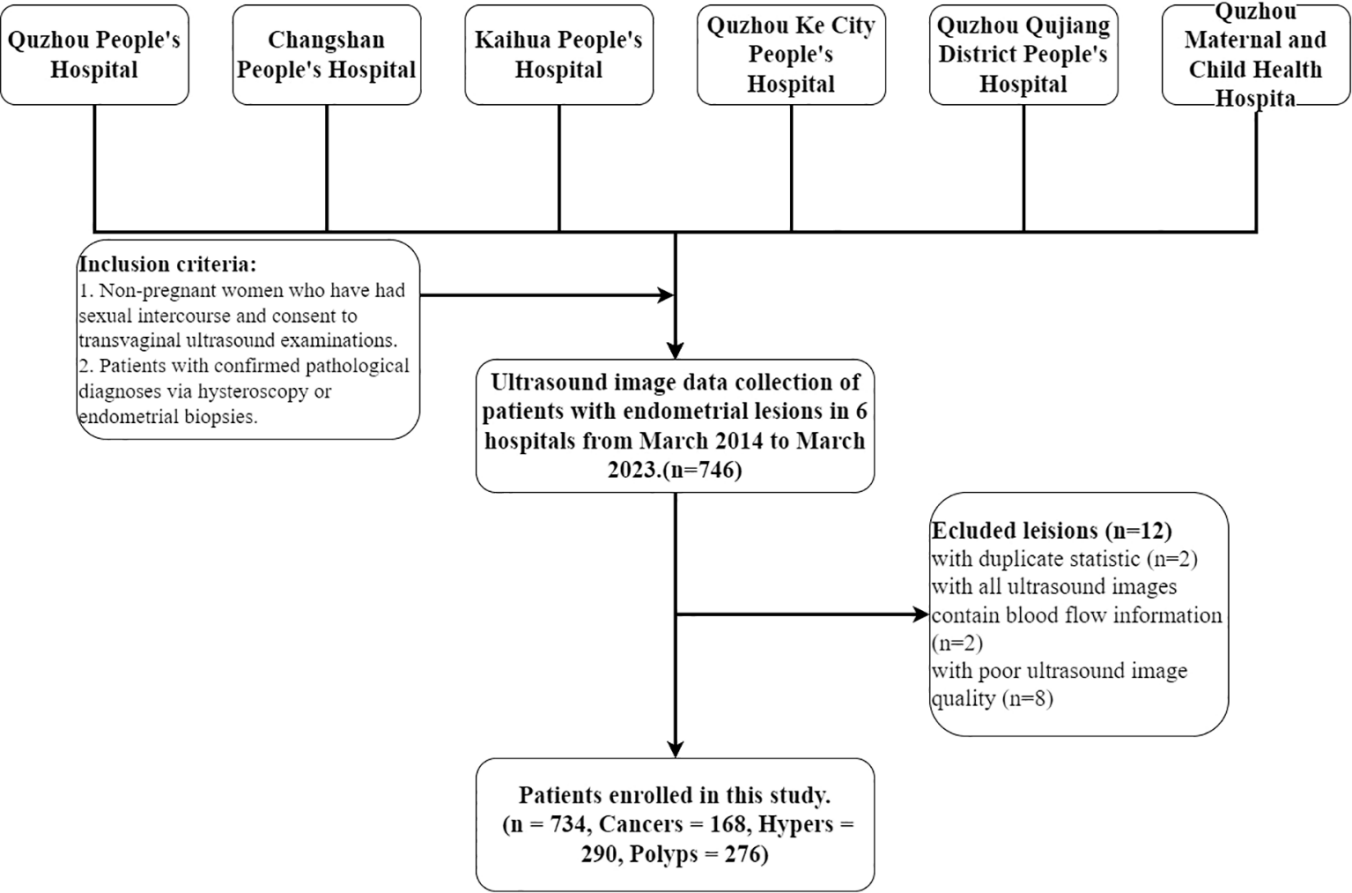
Patient selection workflow. A total of 746 patients with endometrial lesions were collected, of which 734 were ultimately included in the analysis. Cancers, Hypers, and Polyps indicate patients with these types of lesions.

This multicenter retrospective diagnostic study was conducted in line with the principles of the Declaration of Helsinki. This study was approved by the Ethics Committee of the People’s Hospital of Quzhou City (No. 2022-148). Ultrasound examination images were collected from March 2014 to March 2023 at six hospitals: The Quzhou Affiliated Hospital of Wenzhou Medical University, Changshan County People’s Hospital, Kaihua County People’s Hospital, People’s Hospital of Quzhou Kecheng, The Second People’s Hospital of Quzhou, and Quzhou Maternal And Child Health Care Hospital. Inclusion criteria: 1. Non-pregnant women who have had sexual intercourse and consent to transvaginal ultrasound examinations. 2. Patients with confirmed pathological diagnoses via hysteroscopy or endometrial biopsies. Exclusion criteria: 1) Patients who have not had sexual intercourse and are thus ineligible for transvaginal ultrasound examinations. 2) Patients are allergic to condoms and thus unsuitable for ultrasound examinations. 3) Patients with severe reproductive system abnormalities or acute inflammation who are contraindicated for transvaginal ultrasound examinations. 4) Patients with severe psychological disorders who are unsuitable for transvaginal ultrasound examinations. 5) Each patient’s endometrial ultrasound images are collected in two views: all longitudinal images and all transverse images for each case. 6) Image blurring due to significant visual losses and damages during the collection process, along with interferences like gas and artifacts. All images were collected by professional radiologists, and saved in DICOM format. Then, the ultrasound images are further screened, as shown in **Figure 1**; 734 patients were ultimately included in the study.

### 2.2 Data processing

After collection, all ultrasound data were converted from DICOM into JPG files using Python for research. Since data were derived from multiple different hospitals, some preprocessing measures were performed on all images for experiments, including manual cropping to retain only the part captured by the instrument and scaling to 224 × 224. Finally, to improve model robustness and generalization ability, data augmentation techniques, including random-cropping, random-flipping, and TrivialAugment (21) were also used during the training phase. In the testing phase, only size adjustment and normalization of the original images were conducted.

### 2.3 Data enhancement framework

**Figure 2:**
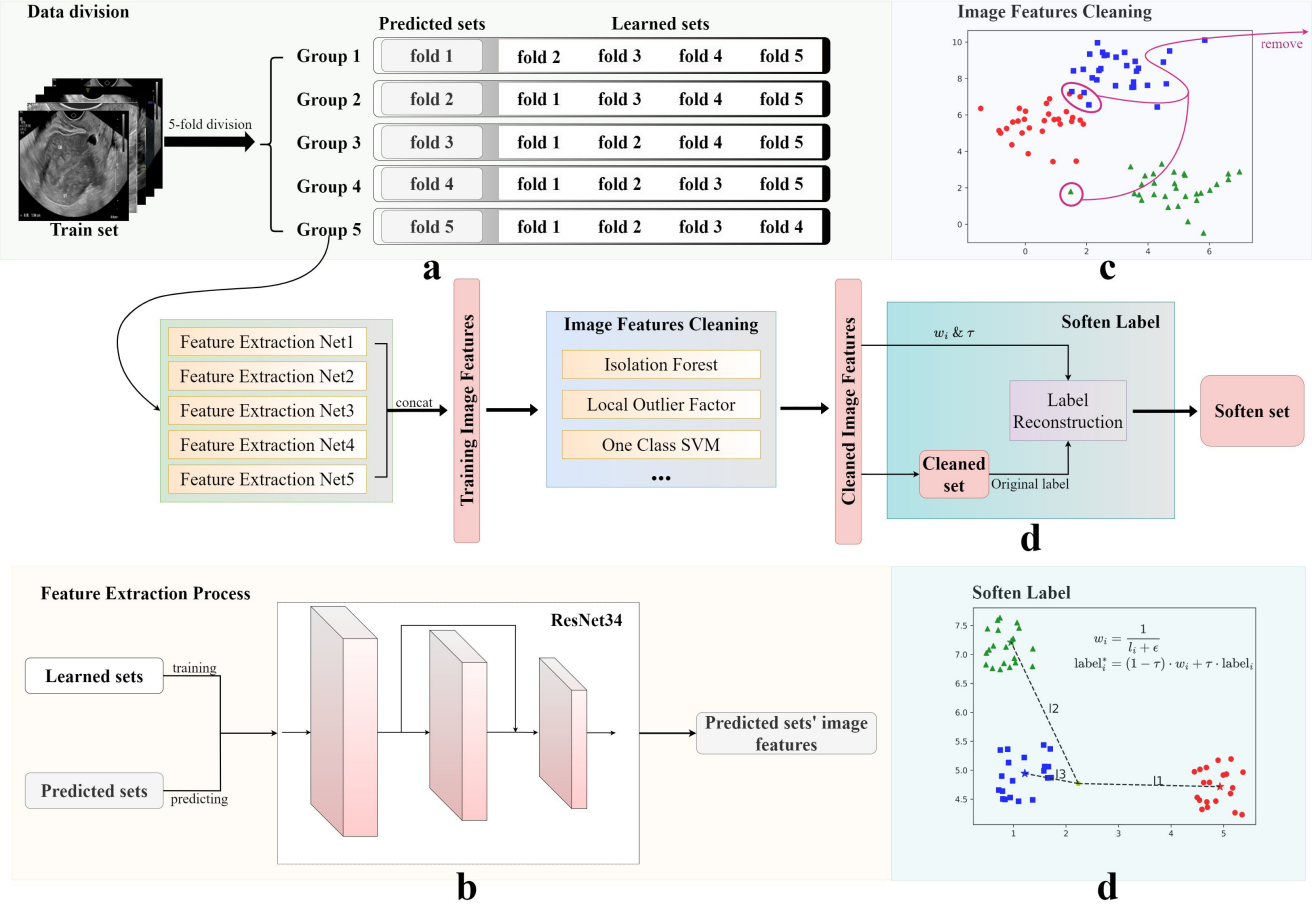
Image features cleaning and soften label processes. The original training set was obtained using four steps: **(A)** data division, **(B)** image features cleaning, **(C)** feature extraction, and **(D)** soften label, to obtain the final soften set. The Soften Label subfigure shows the calculation formula used for softening labels.

An innovative data augmentation framework, primarily encompassing data cleaning and label softening procedures, was developed in this study. The processing of training set data using this framework is summarized in **Figure 2**. Following a feature extraction process, image feature cleaning, and soften label implementation, the training set was utilized to generate a softened set for training purposes.

#### 2.3.1 Image feature cleaning

Medical data are often intricate, encompassing numerous variables and factors, and the diverse types of noise they contain represents a substantial challenge (22). For example, data for the present research was sourced from multiple hospitals, where the process of ultrasound image acquisition is influenced by objective factors, such as equipment performance, environmental noise, and patient size and positioning, which can lead to the presence of abnormal images and noise within the dataset, with potential to impair model performance. To mitigate this possibility, a rigorous data cleaning process was initiated following division of the original data into training and testing sets.

As illustrated in **Figure 2**, five-fold cross-validation was first applied to partition the training set into five subsets, four of which were used to train an independent DL model. These models were primarily tasked with predicting the results from the remaining subsets and generating corresponding image feature vectors. In this study, ResNet34 was used as the backbone network of the framework. Finally, five sets of experimental results were connected to form a complete training set of image features.

Subsequently, anomaly detection methods, such as Isolation Forests (23), were introduced to analyze the feature vectors of the generated training set and exclude potential anomalous data. The training sets selected by three methods were then merged to form a new, cleaned training set. In this study, we selected Isolation Forest, Local Outlier Factor, and One-Class SVM. The selection of methods is contingent upon the data and the specifics of the research. This innovative approach to data cleaning ensured the robustness of the developed model, despite the diverse and potentially noisy data sources.

#### 2.3.2 Soften label

To enhance generalization ability of the model and alleviate overfitting, a label smoothing strategy was implemented, based on the inverse proportion of image-to-cluster center distance. As shown in **Figure 2**, Soften Label included the following processes: first, dimension reduction and clustering were performed on the new processed training set; then, the center of each category cluster and the distance of each image to each cluster center were calculated; finally, new labels were formed, according to the distance ratio. In addition, an adjustable temperature, τ , was introduced, to control the smoothness of the label. The new label for training was obtained by calculating the inverse distance ratio multiplied by τ , plus the hard label value. Datasets were named at different processing stages as the cleaned set and the softened set.

### 2.4 Model architecture and training strategy

**Figure 3:**
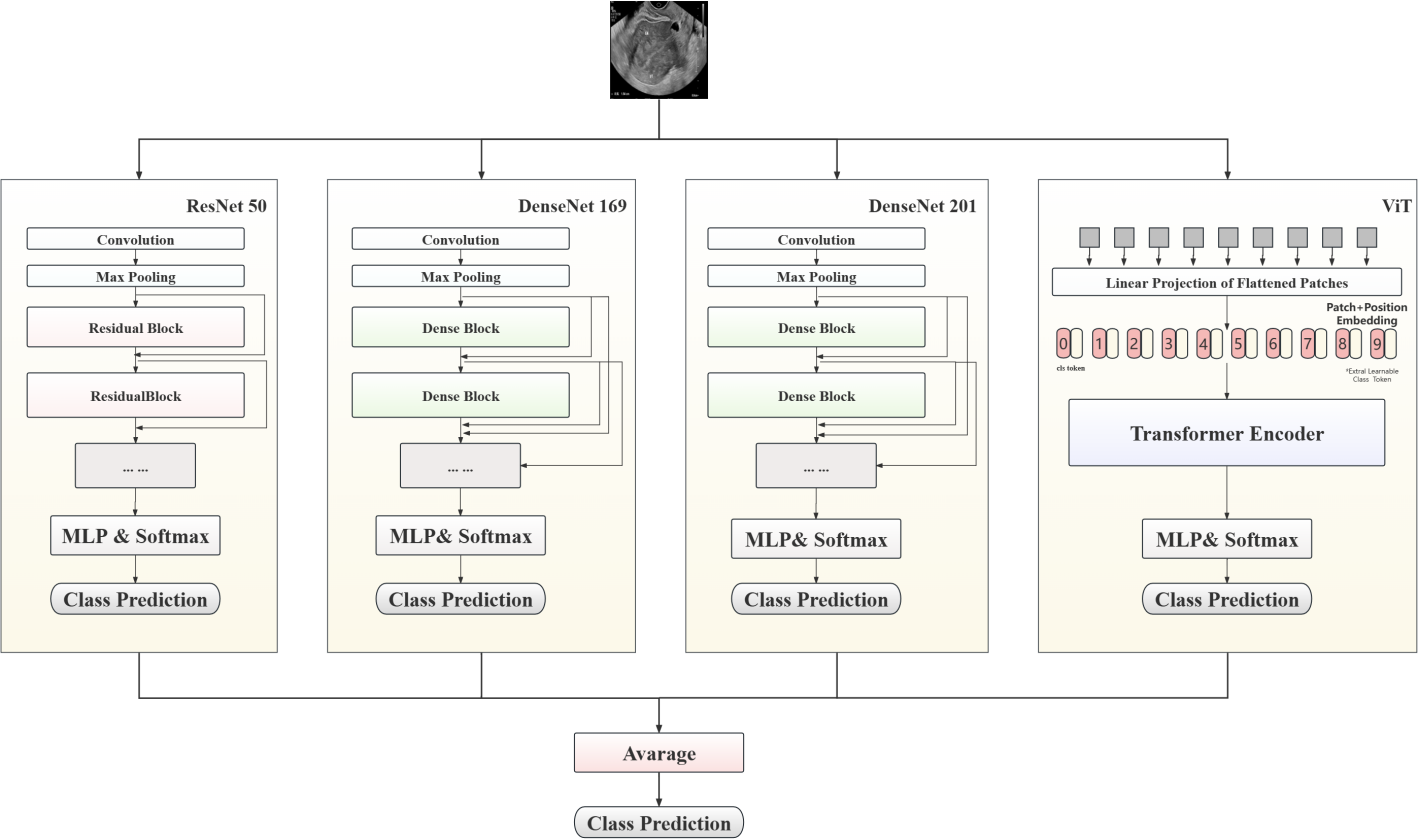
Architecture of model integrating ResNet50, DenseNet169, DenseNet201, and ViT-B.

In this study, a hybrid model to predict patient lesion types, based on convolutional neural network (CNN) and Transformer architectures, is proposed, with the aim of maximizing prediction accuracy. As shown in **Figure 3**, the proposed model combines three classic CNN models (ResNet50, DenseNet169, and DenseNet201) and ViT-B, leveraging the complementary strengths of these different models to enhance the accuracy of endometrial ultrasound image classification.

The multilayer perceptron layer of the original model was tailored to suit this classification task. Each preprocessed image was fed into the model for automatic processing, outputting a three-dimensional array. After Log-Softmax function processing, the prediction probability for each image was obtained. During model integration, the prediction probabilities from all sub-models were weighted to yield the final result. In the testing phase, the average prediction probability for all images from a single patient was calculated, to determine the prediction result.

The experiment comprised three stages. Initially, unmodified ResNet50 was employed as the base model and the impact of different data processing methods on model performance assessed. Subsequently, the applicability of the proposed method was explored by training various CNN and visual transformer models, and the results statistically analyzed after setting the τ value. Finally, high-performing models from the second stage were integrated to test the performance of the optimal model. During the training process, CrossEntropyLoss was used as the objective function, and AdamW was used as the optimizer for end-to-end training. Additionally, the transformer architecture network was loaded with pretraining parameters.

In this manuscript, suffixes have been added to indicate different models; for example, ResNet50_b represents the baseline model, while ResNet50_c represents the model trained using the cleaned set data. Similarly, models trained using softened set data have the suffix “_s”.

### 2.5 Devices and software

This was a multicenter study, and different hospitals used various devices for the data collection process, including Samsung WS80A, GE Volkswagen E10, GE Volkswagen E8, PhilipPsQ5, PhilipPsQ7, and Mindray Resona 6s. All equipment met the experimental requirements. The protocols for each scanning instrument are shown in **Table 1**.

**Table 1:** Scanning Instrument Protocol.

	GE Voluson E8	GE Voluson E6	mindray Resona 7s	mindray Resona 6s	PHILIPS EPIQ-7	PHILIPS EPIQ-5
**Intracavitary probe**	RIC5-9	IC5-9-D	V11-3HU	DE10-3U	3D9-3V	C10-3V
**Probe frequency**	5-9MHz	5-9MHz	3-11MHz	3-10MHz	3-9MHz	3-10MHz
**Bandwidth**	4.5-9.8MHz	4.5-9.8MHz	2.5-12.2MHz	2.8-11.8MHz	2.7-9.2MHz	2.8-1.2MHz
**TIS**	0.4	0.4	0.3	0.3	0.3	0.4
**Depth**	6.0cm	7.0cm	7.0cm	8.0cm	7.0cm	6.0cm
**Magnification**	1.2	1.5	1.1	1.1	1.1	1.1
**Maximum fan angle**	180°	180°	180°	180°	180°	180°
**Frame rate**	40HZ	41HZ	42HZ	42HZ	49HZ	47HZ
**Gain**	40%-80%	40-70%	40-70%	40-70%	40-70%	40-70%
**Dynamic range**	50-120	50-120	50-120	50-120	50-120	50-120

### 2.6 Statistical analysis

Statistical analyses were performed during the testing phase, with individual cases serving as the smallest unit of measurement. Models were validated on a test set, followed by statistical evaluation of the confusion matrix derived from the validation outcomes. Additionally, receiver operating characteristic (ROC) curves were plotted. Primary indicators for comparing model performance were accuracy and area under the ROC curve (AUC); sensitivity and specificity were also considered as indicators of the classification capabilities of models. Two visualization techniques, Grad-CAM (24) and t-SNE (25), were employed to elucidate the operational mechanism of the model.

## 3 Results

### 3.1 Case inclusion and grouping

Among 1875 high-quality images from 734 patients, we randomly extracted 30% of cases as a test set. The remaining images were used as the original training set for data augmentation and model training. The detailed dataset partitions used in this study are presented in **Table 2**. All experiments were trained and tested using the same data-division. Our final model achieved the best performance, with accuracy and macro-AUC values of 0.809 and 0.911, respectively.

**Table 2:** Partition details of the endometrial lesion classification dataset.

Category	Datasets		Training set		Testing set	
	Patients	Images	Patients	Images	Patients	Images
**Cancer**	168	460	118	323	50	137
**Hyper**	290	661	203	470	87	191
**Polyp**	276	746	193	506	83	240
**Total**	734	1867	514	1299	220	568

### 3.2 Impact of innovative strategies

In the methods testing phase, we chose ResNet50 as the baseline model. Model performance was significantly improved through feature cleaning and soften label processing. As shown in **Table 3**, when the original training set was used for training, the accuracy of the test set was only 0.691. This provided us with a comparison baseline; the baseline was determined in the same way for each model in subsequent multi-model comparisons. We noticed that abnormal images in the training set could affect model training; therefore, we used feature cleaning to reprocess the training set. After obtaining relatively clean data, the accuracy of the model on the test set increased to 0.741. In subsequent experiments, we used a label-softening method to reconstruct the labels in the new dataset. Under the same data augmentation and image preprocessing, the accuracy of the model increased to 0.764. The independence and invariance of the test set were ensured in each training batch.

**Table 3:** Impact of different data processing approaches on model performance.

Dataset	Model	ACC	AUC	F1	Recall	Precision
**Base**	ResNet50	0.691	0.811	0.680	0.665	0.697
**Cleaned set**		0.741	0.845	0.736	0.728	0.744
**Soften label**		**0.764**	**0.873**	**0.752**	**0.745**	**0.759**

Boldface numerals are utilized to underscore the optimal results in this group's trial.

Label smoothness was controlled using the parameter, τ , which is similar to the smoothing coefficient in Label Smooth (26). In this experiment, we introduced a variety of different τ values, to generate different soften-labels. ResNet50 showed different classification capabilities under different values of τ. As shown in **Table 4**, ResNet50 performed best when τ was 0.7. To further study the impact of τ on model training, we introduced five other models, including DenseNet169, DenseNet201, EfficientNetB4, VGG16-bn and ViT-B. As shown in **Table 4**, our framework effectively improved the representation learning of various models, indicating that the improvement in the performance of ResNet50 was not isolated. Further, the best performance of each model corresponded to different values of τ. Among individual models, DenseNet201 achieved the best accuracy when τ was 0.9. When τ was 0.7, the performances of ResNet50, DenseNet169, and VGG16-bn were better than those achieved with other softening coefficients. These conditions may indicate that the optimal value of τ may vary depending on the characteristics of the dataset, model, and study.

**Table 4:** Model performance comparison (Accuracy).

Model	Base	Soften label (τ)			
		0.6	0.7	0.8	0.9
**ResNet50**	0.691	0.727	0.764	0.714	0.718
**DenseNet169**	0.727	0.755	0.782	0.736	0.736
**DenseNet201**	0.731	0.764	0.745	0.75	**0.786**
**EfficientNetB4**	0.672	0.7	0.69	0.714	0.745
**VGG16-bn**	0.682	0.732	0.745	0.695	0.727
**ViT-B**	0.736	0.782	0.723	0.759	0.75

Boldface numerals are utilized to underscore the optimal results in this group's trial.

### 3.3 Prediction model performance

**Figure 4:**
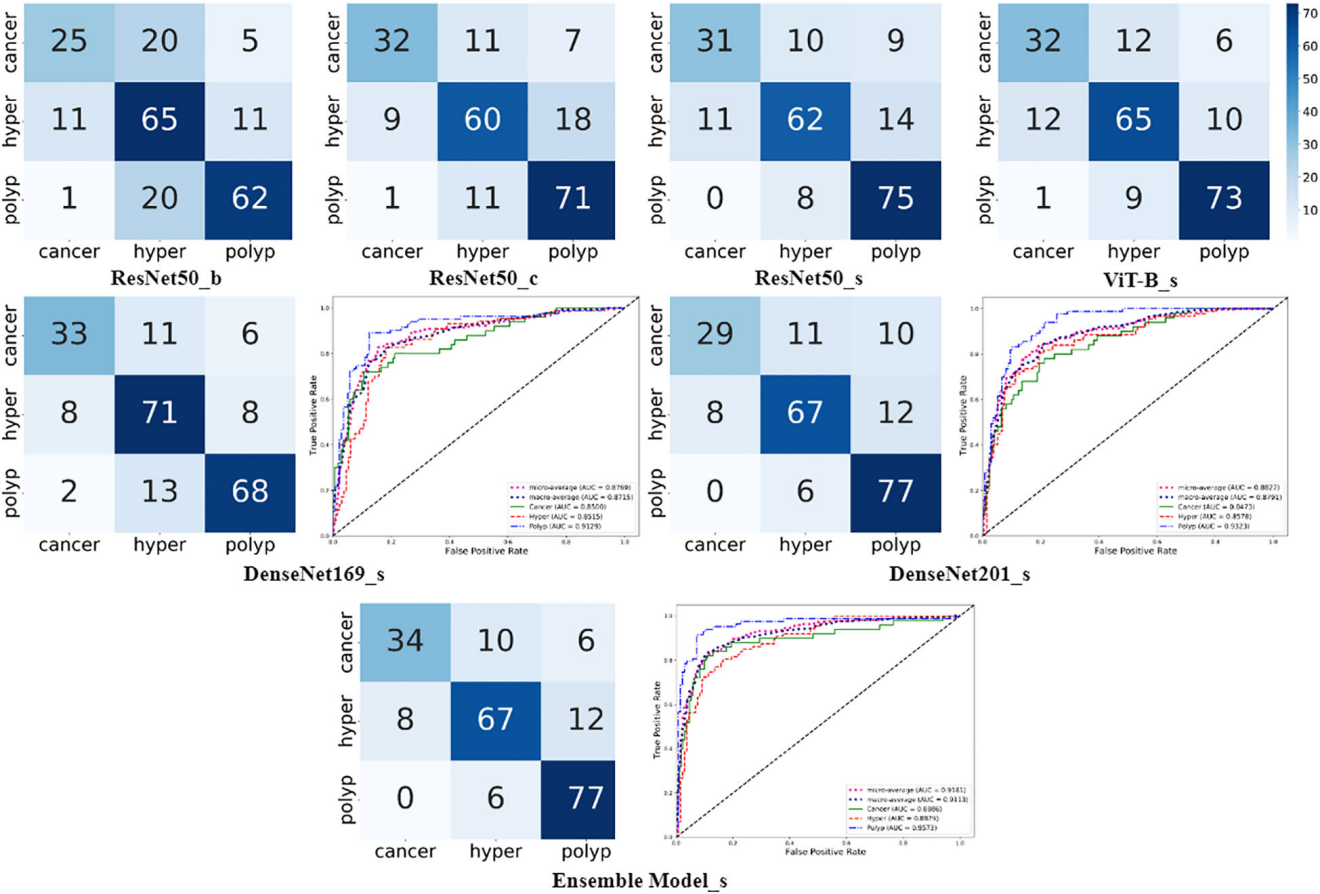
Charts summarizing statistical analysis of results from seven different models. Matrix diagrams represent confusion matrices, while the line plots are ROC curves.

As shown in **Figure 4**, the confusion matrixes for each model effectively reflected their classification performance. In terms of overall accuracy, the DenseNet201_s model exhibited outstanding performance, achieving a best score of 0.786, particularly in recognition of polyp class images, for which it had the best single-category recall rate. We also plotted ROC curves for DenseNet169_s and DenseNet201_s, to evaluate and compare their performances by measuring AUC values (**Figure 4**). We found that DenseNet-201_s was the single model with the best comprehensive classification performance in this study.

In the final phase of our experiment, we implemented an ensemble model approach to enhance the performance of our model. The ensemble models were constructed based on the performance ranking of models as indicated in **Table 4**. As demonstrated in **Table 5**, the Ensemble Model2, which is comprised of ResNet50_s, DenseNet169_s, DenseNet201_s, and ViT-B models, yielded the most superior test results, achieving an accuracy of 0.809 and a macro-AUC of 0.911. As illustrated in **Figure 4**, the Ensemble Model2 outperforms DenseNet201_s in the classification of cancer and hyperplasia. The macro-AUC value of the Ensemble Model2 has significantly improved, and the ROC curve is also more reasonable.

**Table 5:** Performance of ensemble models with different compositions.

Model	Model Composition	ACC	AUC
**Ensemble model1**	**DenseNet169+DenseNet201+ViT-B**	0.777	0.898
**Ensemble model2**	**Ensemble model1+ ResNet50**	**0.809**	**0.911**
**Ensemble model3**	**Ensemble model2+** **EfficientNetB4**	0.805	0.908
**Ensemble model4**	**Ensemble model2+ VGG16-bn**	0.791	0.906
**Ensemble model5**	**Ensemble model3+ VGG16-bn**	0.782	0.912

Boldface numerals are utilized to underscore the optimal results in this group's trial.

### 3.4 Model visualization

The operation process of DL models is often viewed as a ‘black box’ prediction; however, we applied the Grad-CAM and t-SNE visualization methods to explain the working mechanism used by our DL model.

**Figure 5:**
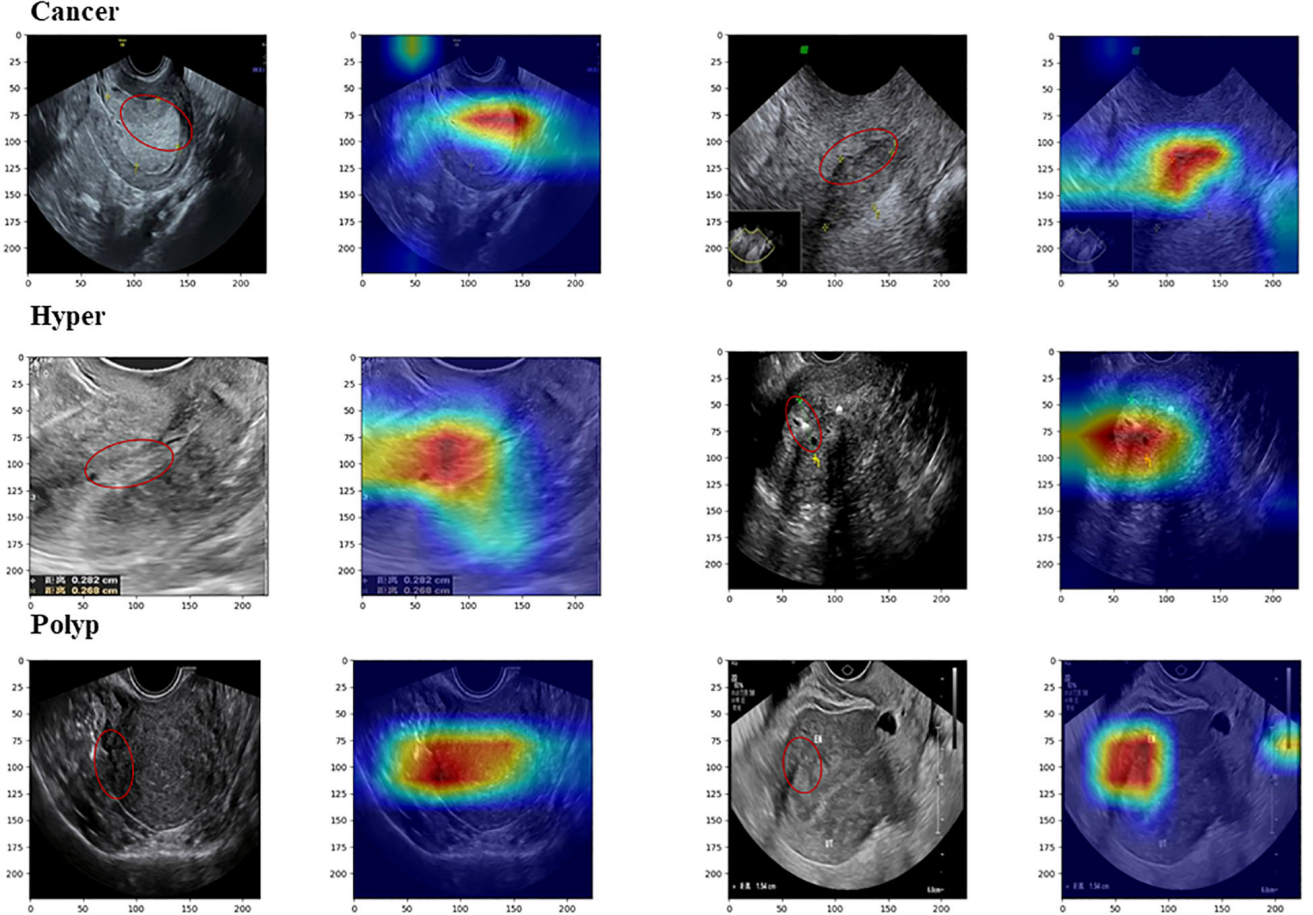
Images from Grad-CAM analysis. The red annotation on the original image indicates the model’s focal region, which closely coincides with the critical lesion area.

In Grad-CAM, we used hook functions to generate the gradient of the last dense module of the model and stacked these gradients onto the original image to generate heat maps. As shown in **Figure 5**, the areas of interest for the model can be distinguished by depth of color. From these images, it can be observed that the model accurately focused on lesion areas in the endometrium; more attention was paid to these areas, and these local features deeply affected model prediction.

**Figure 6:**
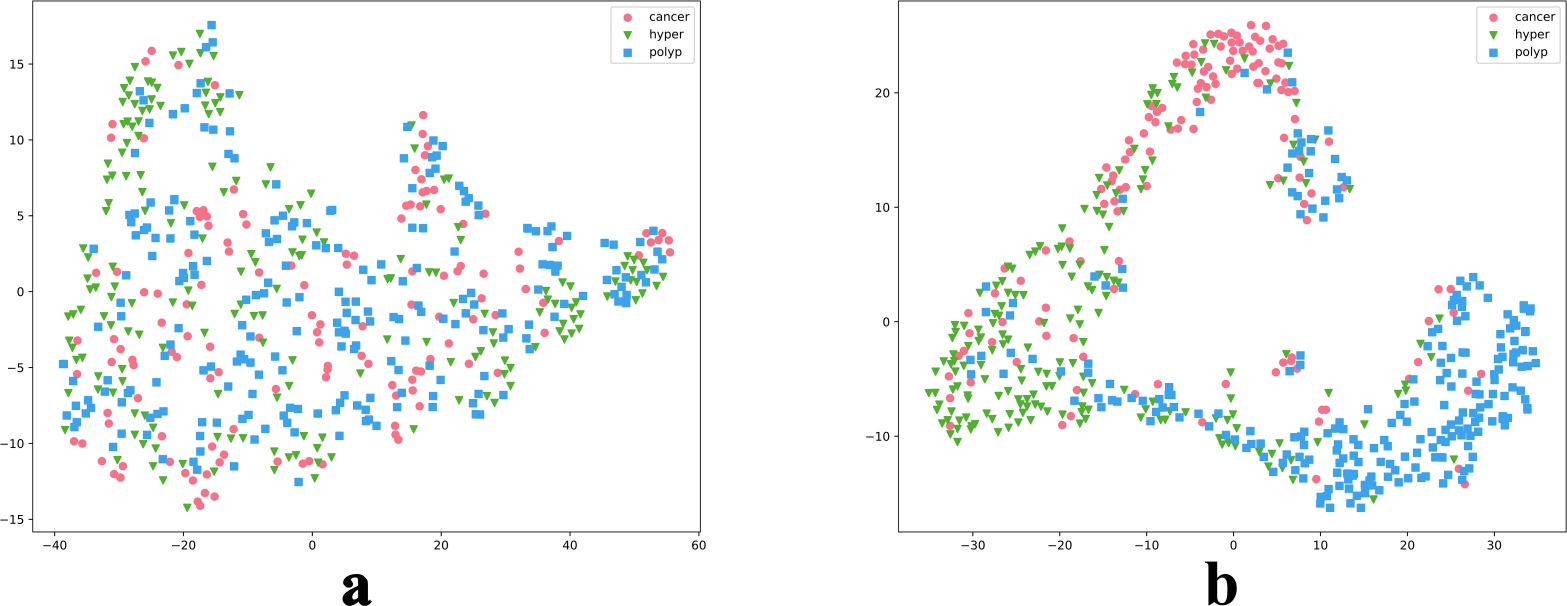
t-SNE reduction of model data. Parts a and b are t-SNE plots before and after model training, respectively.

We also intuitively observed the training effect of the model using the t-SNE method to count the feature vectors extracted by the model. In the high-dimensional space of feature vectors, we calculated the similarities between each data point and mapped these data to low-dimensional space for visualization, and compared the clustering diagrams before and after model training (**Figure 6**). As illustrated in **Figure 6**, most images were mapped in their fixed areas through training, but there was overlap among certain categories. Further, the distance between different category cluster centers reflected the intrinsic relationship of their key image features to a certain extent. We proposed a soften-label method based on this principle.

## 4 Discussion

In the burgeoning field of DL, our study represents a pioneering effort to accurately classify endometrial lesions in ultrasound images using DL models. We achieved an automatic classification with a final accuracy of 0.809 and a macro-AUC value of 0.911.

To maximize DL model effectiveness, we established an innovative data augmentation framework. In this study, collection of datasets from multiple centers ensured inclusion of diverse endometrial lesion ultrasound data. Although this diversity ensured the generalization performance of the model, it also introduced additional noise, which is an inherent challenge commonly present in medical datasets. Within our data augmentation framework, we implemented a scalable data cleaning process, including selection of appropriate feature extraction networks and anomaly detection methods, which significantly improved the accuracy of ResNet50 on our test set, from 0.70 at baseline to 0.741. Another challenge arose from the low signal-to-noise ratio of ultrasound images and the similarity of lesion image features. To address this, we incorporated a label softening strategy, based on clustering and inverse distance, into the data augmentation framework. This strategy, which did not introduce additional prior knowledge, bolstered the model’s understanding of the relationships among lesion image features, thereby improving its generalization and robustness. Consequently, the accuracy of ResNet50 on the test set improved to 0.764, effectively enhancing the fine-grain level of the model. Finally, we integrated multiple distinct DL models, leveraging their respective strengths to improve testing accuracy to 0.809.

In the second stage of our experiment, we applied our method to multiple models, each of which showed significant improvement over their baseline performance. These findings underscore the effectiveness and wide applicability of our approach. In the label softening process, we utilized τ to manage the degree of label softening. Performance of the models varied under different τ values, with each model achieving substantial improvements over their baseline performances under specific τ values; however, the optimal τ value varied across models. Nevertheless, it is difficult to draw clear conclusions based on these findings, for to two potential reasons: first, the limited range of τ values used in the experiments leaves open the possibility that there may be an optimal τ value in other ranges that could yield the best results for the majority of models; and, second, the inherent variations in the architectures of each model could result in varying sensitivities to τ value, leading to differences in optimal τ values among models.

In contrast to previous studies, our research has made significant strides in the classification of endometrial lesions using DL methods to analyze ultrasound images. Unlike prior works that focused on endometrial thickness measurement based on ultrasound images, we have successfully developed a model that can accurately classify endometrial lesions. By integrating innovative strategies, such as feature cleaning and label softening, our model can effectively learn and utilize various ultrasound image features. Based on the findings of Reznak et al., our model achieved better results than medical staff, particularly in the detection of polyps. Consequently, our model significantly enhances endometrial lesion classification accuracy, marking a substantial breakthrough in the field of DL applied to ultrasound-based diagnosis.

Despite these advances, our research has limitations. Our dataset, although diverse, was not sufficiently large, comprehensive, or representative, posing challenges in terms of distinguishing features of endometrial cancer from those of endometrial hyperplasia. Further, during the data collection process, there was a lack of uniform standards among operators. Furthermore, the process involved subjective selection of representative ultrasound images for preservation by operators, which could lead to discrepancies between the knowledge encapsulated in ultrasound image data and real-world conditions (27, 28). This unilateral learning from disparate images may result in suboptimal model performance. To mitigate this issue, we could consider methods akin to those used for the analysis of hysteroscopy or MRI datasets. During the data collection process, comprehensive and continuous data is gathered for each patient. As shown in Yasaka K et al.’s research (29), continuous image data can provide more comprehensive and in-depth information.

For future work, we aim to refine our methods further. We will consider using other models when extracting image features, or even combining additional different models to complete the task. We will conduct further comparative experiments, to determine a more suitable combination of anomaly detection methods. Moreover, we will explore setting of an adaptive τ value, which is currently highly individualized, to further optimize the performance of our model. Despite its limitations, our study has opened up new possibilities for application of DL in medical image diagnosis and provides a crucial reference that can inform future research.

## 5 Conclusion

In this study, we developed a novel DL model that can accurately classify endometrial lesion ultrasound images. This model, enhanced by our innovative feature cleaning and soft label strategies, outperforms traditional models, providing clinicians with more precise diagnostic information. This is the first application of DL in this area and demonstrates its potential value, despite some limitations in data scale and collection. Our research paves the way for future use of DL in medical image diagnosis, particularly as we plan to incorporate more continuous medical imaging data.

## Data Availability

The original contributions presented in the study are included in the article/supplementary material. Further inquiries can be directed to the corresponding authors.
